# Gut Microbiota, Fusobacteria, and Colorectal Cancer

**DOI:** 10.3390/diseases6040109

**Published:** 2018-12-11

**Authors:** Dervla Kelly, Liying Yang, Zhiheng Pei

**Affiliations:** 1 Graduate Entry Medical School, University of Limerick, Limerick, Ireland; dervla.kelly@nyumc.org; 2 Department of Pathology, New York University School of Medicine, New York, NY 10016, USA; 3 Department of Medicine, New York University School of Medicine, New York, NY 10016, USA; Liying.Yang@nyumc.org; 4 Department of Veterans Affairs New York Harbor Healthcare System, New York, NY 10010, USA

**Keywords:** fusobacteria, colorectal cancer, carcinogenesis, microbiome

## Abstract

The gut microbiota has emerged as an environmental contributor to colorectal cancer (CRC) in both animal models and human studies. It is now generally accepted that bacteria are ubiquitous colonizers of all exposed human body surfaces, including the entire alimentary tract (5). Recently, the concept that a normal bacterial microbiota is essential for the development of inflammation-induced carcinoma has emerged from studies of well-known colonic bacterial microbiota. This review explores the evidence for a role of fusobacteria, an anaerobic gram-negative bacterium that has repeatedly been detected at colorectal tumor sites in higher abundance than surrounding histologically normal tissue. Mechanistic studies provide insight on the interplay between fusobacteria, other gut microbiota, barrier functions, and host responses. Studies have shown that fusobacteria activate host inflammatory responses designed to protect against pathogens that promote tumor growth. We discuss how future research identifying the pathophysiology underlying fusobacteria colon colonization during colorectal cancer may lead to new therapeutic targets for cancer. Furthermore, disease-protective strategies suppressing tumor development by targeting the local tumor environment via bacteria represent another exciting avenue for researchers and are highlighted in this review.

## 1. Introduction

There are an estimated 142,820 new cases of colorectal cancer (CRC) annually in the United States, with over 50,000 deaths [1]. Researchers have established the major risk factors of CRC. American males have 25% higher risk of CRC than females, and African Americans have 20% higher incidence than Caucasians. Age is also a risk factor, with less than 10% of cases occurring under the age of 50 [2].

Colorectal carcinomas are classified by etiology as inherited (e.g., hereditary nonpolyposis colorectal cancer due to genetic instability and familial adenomatous polyposis (FAP) coli due to a mutation in the adenomatous polyposis coli gene, APC), inflammatory (e.g., Crohn’s disease and ulcerative colitis), or sporadic [2]. Over 80% of CRCs are classified as sporadic CRC and these have a poorly defined etiology. Sporadic tumorigenesis is thought to involve mutations in the APC (5q), DNA hypomethylation, and multiple epigenetic changes, notably in KRAS2 (12p), DCC (18q), and p53 (17p) [3].

As well as genetic factors, there are environmental factors that increase the risk of CRC. In particular, diet has been associated with CRC. Previous studies have established that consumption of red and processed meats, highly refined carbohydrates, and alcohol carry an increased risk of CRC [4].

The gut microbiota has emerged as an environmental promoter of CRC in both animal models and human studies, which is the focus of discussion from here onward. It is now generally accepted that bacteria are ubiquitous colonizers of the human body, including along the gastrointestinal tract [5]. These bacterial communities are established at birth, and a lifelong symbiotic relationship forms, with the human providing nutrients. In return, bacteria are involved in many processes, including tempering immune responses, metabolizing food and byproducts, and preventing pathogenic bacterial diseases. The concept that normal bacterial microbiota plays a role in the development of inflammation-induced cancer has gained prominence from the considerable colonic microbiota literature. 

## 2. Microbiome and Colorectal Carcinogenesis

Despite large interpersonal variability, it is known that an average colorectal microbiota includes anaerobic bacteria, including *Bacteroides*, *Eubacterium*, *Bifidobacterium*, *Fusobacterium*, *Peptostreptococcus*, and *Atopobium* [6,7]. Facultative anaerobes include *Lactobacilli*, *Enterococci*, *Streptococci*, and *Enterobacteriaceae* are also usually present at about 1000-fold lower abundance [6]. However, an individual’s microbiota is influenced by diet, age, gender, and ethnicity, and its dynamic nature makes it difficult to characterize [7]. From the 1990s onward, studies emerged demonstrating associations between colon cancer and specific bacterial species found in fecal or mucosal tissue samples, including *Streptococcus bovis*, *Bacteroides*, and *Clostridia* [6,8,9]. 

Chen et al. undertook 16S metagenomic profiling to characterize the microbiota present in the intestinal lumen and mucosa of patients with CRC compared to healthy controls. *Bifidobacterium*, *Faecalibacterium*, and *Blautia* were attenuated in CRC patients, whereas *Porphyromonas* and *Mogibacterium* were increased [10]. In the lumen, *Erysipelotrichaceae*, *Prevotellaceae*, and *Coriobacteriaceae* were increased in CRC patients. This suggests that intestinal lumen microbiota may interact with the host to increase CRC risk [10].

In a study by Shen et al., sequencing 21 adenoma and 23 nonadenoma subjects showed enriched *Proteobacteria* and reduced *Bacteroidetes* in cancer tissue [11]. Sobhani et al. analyzed stool bacterial DNA using PCA and found *Bacteroides/Prevotella* species to be more abundant in cancer patients than in control subjects [12]. These sequencing studies have demonstrated the occurrence of gut microbiota alterations in CRC. 

*Fusobacteria*, anaerobic gram-negative rods, are rare agents of severe human diseases [13] that have recently been the center of academic debate after researchers repeatedly noted their link to CRC. *F. nucleatum* (Fn) and *F. necrophorum* are the commonly encountered members of the *Fusobacterium* species. They commonly inhabit the oral cavity, occasionally causing periodontal and gingival infections [14]. The rest of the chapter explores the evidence for the role of *Fn* in CRC.

## 3. Clinical Significance of Fn in CRC

Fn buildup in colorectal carcinoma tissue compared to healthy gut tissue was first reported in 2011 by two research groups [15,16]. They found that the abundance of Fn in CRC was significantly higher than in histologically normal tissue adjacent to the tumor. The difference is also evident in stool samples [17,18]. Fn enrichment has also been observed in colorectal adenoma, the precursor of CRC [18,19]. This has led to fecal Fn measurement being touted as a useful predictive marker in the clinical management of CRC [20].

Several metagenomics-sequencing studies have shown that increased Fn abundance was positively associated with CRC mortality. For example, one study found CRC patients with high fusobacterial levels had significantly lower overall survival than patients with average levels of Fn (*p*  =  0.008) [18]. This would suggest that the enrichment of Fn in CRC tissue could serve as a prognostic biomarker [18,20,21]. Studies have also demonstrated a positive association between increased amounts of Fn and CRC metastases, such as hepatic metastatic disease [15,20,22]. These findings indicate that Fn-high CRC maybe be a clinically relevant subtype of CRC that promotes tumor progression. However, several studies did not identify an association between Fn and CRC prognosis [23,24,25]. There are still many gaps in our understanding of the role of Fn in CRC and, hence, Fn as a prognostic marker.

## 4. Translocation of Fn from the Oral Cavity 

How Fn colonizes the colon from the mouth is not yet known. Several studies have reported that microbial colonizers of the oral cavity predict the microbial composition of the gut and vice versa [26,27]. This suggests the oral cavity may act as a repository for Fn, which is then swallowed to the gut. 

Fn is a major cause of periodontal disease, but epidemiological studies linking periodontal disease and CRC have been inconclusive. Momen-Heravi et al. (2017) described the increased risk of CRC in a large cohort study [28], while a separate study found no association between the amount of Fn in the oral cavity and CRC [29].

Meyerson et al. (2017) showed that Fn colonization of CRC is conserved in nonadjacent liver metastases, indicating microbiome similarities between paired primary–metastatic tumors [30]. This suggests that Fn might form a symbiotic association with the tumor during metastasis via lymphatic or hematogenous pathways.

There is also uncertainty about where Fn is found within the colon. Notably, Fn has been observed principally in proximal colon cancer, with moderately increasing proportions of Fn-high CRC detected on the right side, from rectum to cecum [24,31]. However, this effect was not seen in other experiments [32]. These observations are complicated by the fact that right colon cancer exhibits mucus-invasive bacterial biofilms, which can display blooms of Fn [32].

Overall, there are no conclusive data to support the hypothesis that the origin of the tumor-associated fusobacteria is as passengers from the oral cavity rather than residential bacteria in the colon. 

## 5. Molecular Mechanistic Pathways Associated with in Fn-Positive CRC

**Figure 1:**
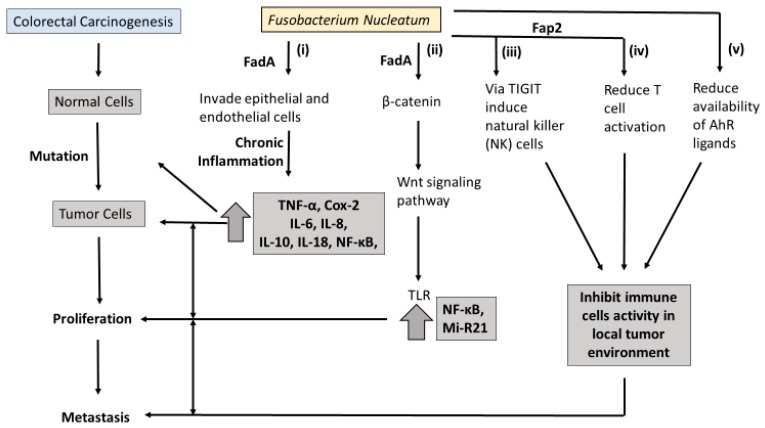
Summary of five hypothetical pathways of Fusobacterium nucleatum (Fn) in promoting colorectal carcinogenesis: (i) FadA in Fn promotes chronic Fn infection of colon mucosa that triggers reactive oxygen species (ROS) and proinflammtory mediators; (ii) Fad2 activates β- catenin signaling that, through the Wnt signaling pathway, results in increased miRNA and NF-κB, and ultimately increases tumor proliferation; (iii) Fap2 interacts with TIGIT on natural killer (NK) and T cells to suppress immune response and promote tumor growth; (iv) Fn through Fap2 may recruit tumor-infiltrating myeloid cells; (v) Fn in conjunction with other microbiota may affect the production of Aryl hydrocarbon receptor (AhR) ligands. AhR mediates the transcription of cytokines that regulate immune response and immune-response inhibition promotes tumor growth.

Molecular pathology offers an opportunity to understand how Fn may contribute to the carcinogenesis of CRC. An overview of the proposed pathways linking Fn to colorectal carcinogenesis is presented in Figure 1. At least three molecular pathways have been described in colorectal-cancer initiation and progression. The chromosomal instability (CIN) pathway is seen in both FAP and sporadic CRC. It involves chromosomal abnormalities such as deletions, insertions, as well as a loss of heterozygosity and KRAS mutations [33]. Hereditary nonpolyposis colorectal cancer (HNPCC) involves the mutator phenotype/mismatch repair pathway with germline mutations in the *hMLH1, hMSH2, hMSH6*, or *PMS2* genes, and also presents with KRAS mutations [34]. 

The third hypermethylation phenotype sees a polyp as the precursor lesion to cancer. Characteristics of the hyperplastic/serrated polyp pathway include genetic alterations, including the hypermethylation of some CpG islands and BRAF mutations [35]. This alteration may result in the hypermethylation of the promoter region of mismatch repair enzymes, such as MLH1 [36]. These are all plausible mechanisms for the development of neoplasms, but data demonstrating the involvement of Fn are limited.

Microsatellite instability (MSI) is a conspicuous pathological feature of CRC [37]. Studies have demonstrated increased MSI in colorectal-cancer tissue with high Fn [24,25,38]. However, the sequence of progression involving MSI and Fn is still unknown. One potential interpretation is that Fn involvement in inflammation pathways recruits inflammatory cytokines and enhances the production of reactive oxygen species (ROS) (Figure 1i). These inflammation/ROS signaling pathways reduce the activity of mismatch-repair (MMR) protein, resulting in MSI.

Another important characteristic of chronic gut mucosal inflammation is the CpG island methylator phenotype (CIMP). CpG island methylation reduces gene expression-suppressing epigenetic events. A number of studies have found Fn to be present in high-CIMP CRC [21,24,38]. Interestingly, Tahara et al. (2014) found Fn-enriched CRC to be correlated with several genetic characteristics, namely wild-type TP53, mutant CHD7/8, and increased somatic mutations. The growing body of research has come to the belief that Fn may be associated with specific molecular subsets of CRC.

## 6. Fn Virulence Proteins and Colorectal Carcinogenesis 

In the last decade, two outer membrane proteins from Fn, Fap2 and FadA, have been implicated in a bacterium-dependent tumor-invasion mechanism. Fn has been shown to invade human epithelial cells, activating β-catenin signaling and promoting the growth of CRC cells via the FadA adhesion virulence factor [39] (Figure 1ii). Gur et al. identified a transporter protein, Fap2 which they believe increases the invasive ability of CRC via suppression of the immune response [40]. It is believed the Fap2 virulence factor directly interacts with the checkpoint receptor TIGIT on natural-killer (NK) cells and lymphocytes, suppressing the antitumor actions of lymphocytes (Figure 1iii,iv).

Binding FadA to vascular endothelial cadherin may enhance beta-catenin signaling and the Wnt signaling pathway, and promote CRC carcinogenesis [39]. Toll-like receptor 4 (TLR4), a critical protein for bacteria lipopolysaccharide (LPS), is upregulated in intestinal inflammation and thought to mediate CRC initiation and progression [41]. Chen et al. found that both Fn and LPS extracted from Fn activate the beta-catenin transduction pathway in colorectal tumor cells via the TLR4/P-PAK1 mediators [42]. 

MicroRNA-21 (miR-21) was enriched in the colorectal tissue and serum of patients with ulcerative colitis and CRC. MiR-21 is a transcriptional modulator of cellular processes such as proliferation, differentiation, and apoptosis, but its link to CRC prognosis is unclear. Studies using Fn-infected colorectal-cancer mouse models have found that FN increases mi-R21 expression by activating TLR4 [43]. Some researchers haves suggested that ligands targeting miR-21 may have a role in inhibiting colitis-associated colon cancer (CAC) [44]. 

In summary, the beta-catenin/WNT transduction cascade seems to have a key role in Fn-mediated colorectal carcinogenesis. Further research is needed to determine how Fn-virulence proteins relate to colorectal carcinogenesis.

## 7. Fn, Inflammation and Immunity in CRC 

Extensive evidence has established the role of inflammation as contributing to and increasing the risk of CRC. Both inflammatory bowel diseases, Crohn’s disease and ulcerative colitis, bring an increased risk of cancer [12]. The complex signal exchange between the diverse members of the gut microbiome and immune system is a challenge that computational biologists are taking on. Schirmer et al. (2016) mapped the inflammatory cytokine production associated with bacteria [45]. 

Several studies using immunoassays in colorectal tissue and in vitro cultures demonstrate that Fn infection increases the expression of inflammatory cytokines in tumor tissue, including IL-6, IL-8, TNF-α, and Cox-2, and these cytokines are capable of promoting tumor development [39,46,47,48]. Ye et al. found that Fn can trigger the release of chemokine CCL20 in colorectal-cancer cells cultured with Fn [49]. Moreover, Fn stimulates macrophage activation, migration, and tumor infiltration [49,50]. NF-κB, a transcription factor thought to have a critical role in tumor growth [51], is raised in Fn-enriched CRC [43,46]. 

Fn, like many human bacterial pathogens, has been linked with immune suppression. Fn suppresses the immune system by inducing cell death in human lymphocytes [50,52,53]. Mima et al. observed inverse association between the amount of CD3+ T-cell density and Fn [25], while Park et al. were unable to find any significant change in CD3+ T-cell density with varying Fn abundance [50]. 

Recent metabolomics studies have identified L-tryptophan (Trp) as an essential amino acid that plays a role in the balance between intestinal immune response and gut-microbiota maintenance. Bacteria in the gut, including Fn, metabolize Trp to indole, tryptamine, and skatole derivatives, with the rate depending on the unique combination of catalytic enzymes present in a given microbiota [54]. They indirectly mitigate endogenous Trp metabolism in the body [55]. Furthermore, variations in Trp metabolism have been shown to alter microbial proliferation and microbiota diversity [56]. Trp metabolites modulate serve as ligands of the Aryl hydrocarbon receptor (AhR), which is believed to partly regulate inflammatory response in gut epithelial tissues. It has been proposed that excessive degradation of AhR ligands results in the loss of control of intestinal immune response [57,58] (Figure 1v). Similarly, intestinal inflammation was attenuated in mice following inoculation with Lactobacillus strains capable of metabolizing Trp to produce AhR ligands [59]. 

Fn in conjunction with dysbiosis of the local gut microbiome is a contributing factor in the proliferation of tumors. Therefore, it has been hypothesized that therapies that target bacteria to modulate immune response are a promising route for antitumor immune therapy. Several recent papers have demonstrated positive immunotherapy responses in people to specific varieties of gut bacteria [60,61,62]. Translating these positive findings into meaningful clinical therapies requires teasing out the mechanisms of the complex biological pathways outlined above. 

## 8. Detection of Fn in CRC 

Several different methods are used to detect Fn. These include conventional quantitative polymerase chain reaction (qPCR), fluorescent quantitative PCR (FQ-PCR), metagenomic sequencing, fluorescence in situ hybridization (FISH), 16S ribosomal RNA sequencing, and droplet digital PCR (ddPCR) [63]. In addition, sample collection methods vary among studies, from formalin-fixed paraffin-embedded CRC tissue, CRC frozen tissue, genomic DNA, and feces collected from CRC patients. Among these detection methods, qPCR is the least expensive and most widely used. 

The original 2011 studies that first demonstrated the association between Fn and CRC both used sequencing-based methods to identify the bacteria. A 2018 replication study detected Fn in just 25% of colorectal carcinomas, and the difference in the level of this species in colorectal carcinomas and adjacent tissue was not significant [64]. The lack of replication may be due to differences in sample size: the replication study had 40 matched cases, while the original Casterellain study had 99 [15]. Technical issues with qPCR might also have contributed to the variation in results [65]. The replication-study results highlight the need for accurate Fn-detection methods, as well as prospective human studies.

## 9. Cause or Consequence

Whether Fn colonization is a cause or consequence of CRC is unknown. Experiments that used daily doses of Fn in rodent models of colon cancer indicate that Fn acts in the early stages of CRC, creating a proinflammatory environment that supports tumor progression [43,46]. However, germfree mouse experiments involving the daily administration of two strains of Fn failed to promote tumorigenesis, and therefore suggest that Fn on its own may not induce either inflammation or cancer [66]. Meanwhile, other studies favor a role for Fn in the late stages of CRC [15,30]. The development of cancer is multifaceted, and the gut microbiome is a contributing factor.

## 10. Summary and Perspectives

The observations of the active role of the gut microbiome in tumorigenesis have resulted in an energetic search for causative agents among normal flora bacteria. This is a plausible hypothesis given the evidence of bacterial roles in chronic inflammatory disorders and related cancers. Studies of genetically deficient rodent models demonstrate an association between Fn and CRC. This review has identified studies that demonstrate the ability of Fn to colonize mucosal surfaces in the colon and invade human epithelial cells, activate beta-catenin signaling, induce oncogenic gene expression, and promote the growth of CRC cells. 

Gut bacteria are implicated in carcinogenesis due to evidence of stimulating inflammatory responses and suppressing host immune reactions to tumor growth. Two Fn surface-proteins, FadA and Fap2, are thought to increase the virulence of Fn by facilitating it entering cells and contributing to gene expression. Furthermore, gut bacterial activation of the WNT signaling pathway, the MSI pathway, and of inflammatory responses in the host at the site of a tumor, suggest a contributory role for Fn in CRC progression and a role of therapies targeting the gut microbiome to modulate the host immune response to tumors. Further investigation of gut-microbiome metabolites is required to tease out their role in complex signaling cascades regulating host immune responses to cancer.

However, the capacity for causing inflammation or cancers may depend on the summative activities of multiple species in any given microbiota rather than on a single species. Another possible explanation is that the host role is critical here. Human gene defects, in collaboration with a normal bacterial microbiota, could in theory lead to cancer development in an individual over their lifetime. Future investigations could seek to discover any relevant weak gene defects in human patients, and correlate them with long-term pathogenesis. Whether Fn enrichment is a consequence or a cause of carcinogenesis or inflammation in colorectal tissue remains to be elucidated. Prospective studies could provide more definitive answers on the temporal order between Fn and CRC. Even if Fn colonization is a consequence of CRC, it may still play an important role in enhancing tumor malignancy, promoting metastasis, and evading antitumor immunity. 

The role of Fn in tumorigenesis has thrown up some intriguing questions about cancer causes. Cancer development could potentially be reduced by manipulating the bacterial microbiota using probiotics, fecal implants, and antibiotic therapies or vaccination. Fecal Fn may be a favorable measurable biological marker for CRC detection, but more research is needed, as it is unclear how it varies throughout the stages of colorectal cancer. Therefore, considerably more work is needed on host–bacteria crosstalk and the virulence proteins that are involved in colorectal carcinogenesis.
